# Robust Feature Representation Using Multi-Task Learning for Human Activity Recognition

**DOI:** 10.3390/s24020681

**Published:** 2024-01-21

**Authors:** Behrooz Azadi, Michael Haslgrübler, Bernhard Anzengruber-Tanase, Georgios Sopidis, Alois Ferscha

**Affiliations:** 1 Pro2Future GmbH, Altenberger Strasse 69, 4040 Linz, Austria; michael.haslgruebler@pro2future.at (M.H.); bernhard.anzengruber@pro2future.at (B.A.-T.); georgios.sopidis@pro2future.at (G.S.); 2 Institute of Pervasive Computing, Johannes Kepler University, Altenberger Straße 69, 4040 Linz, Austria; ferscha@pervasive.jku.at

**Keywords:** human activity recognition, wearable, deep learning, representation learning, multi-task learning, alpine skiing

## Abstract

Learning underlying patterns from sensory data is crucial in the Human Activity Recognition (HAR) task to avoid poor generalization when coping with unseen data. A key solution to such an issue is representation learning, which becomes essential when input signals contain activities with similar patterns or when patterns generated by different subjects for the same activity vary. To address these issues, we seek a solution to increase generalization by learning the underlying factors of each sensor signal. We develop a novel multi-channel asymmetric auto-encoder to recreate input signals precisely and extract indicative unsupervised futures. Further, we investigate the role of various activation functions in signal reconstruction to ensure the model preserves the patterns of each activity in the output. Our main contribution is that we propose a multi-task learning model to enhance representation learning through shared layers between signal reconstruction and the HAR task to improve the robustness of the model in coping with users not included in the training phase. The proposed model learns shared features between different tasks that are indeed the underlying factors of each input signal. We validate our multi-task learning model using several publicly available HAR datasets, UCI-HAR, MHealth, PAMAP2, and USC-HAD, and an in-house alpine skiing dataset collected in the wild, where our model achieved 99%, 99%, 95%, 88%, and 92% accuracy. Our proposed method shows consistent performance and good generalization on all the datasets compared to the state of the art.

## 1. Introduction

HAR is an active field of research in pervasive computing that aims to detect human physical activities through machine learning models. HAR has various applications in healthcare [1,2], sports [3,4], industry [5,6,7], and other fields. Commonly, HAR models utilize activity signals recorded by wearable or visual sensors. While wearable sensors are unobtrusive, low-cost, and portable, vision-based sensors [8] are inflexible, limited to environmental factors [9], and suffer from privacy-related issues [10]. Therefore, wearables such as Inertial Measurement Units (IMUs) have been employed frequently in data recording for HAR applications [11,12,13].

Without regard to the learning approach, HAR consists of four main stages: data acquisition, data preparation, feature learning, and recognition task [14]. Among all the HAR steps, scholars have reported data acquisition and feature learning as major HAR bottlenecks [15,16]. Although publicly available datasets, to some extent, alleviate the former, the latter remains the number one challenge [15], which causes poor generalization. Consequently, the model’s accuracy drops when facing data from unseen subjects [16]. Moreover, the model’s confusion may increase when activities in a dataset are very similar or complex, meaning that one activity is a mixture of the other actions [17], or when users can perform activities in different ways. For instance, one can think of cycling in sitting or standing mode or alpine skiing in different styles.

To overcome such problems, traditionally, scientists tried to extract meaningful features from the IMU signals to train machine learning models. Hand-crafted features are tedious to create, limited, and need domain expertise. As a result, utilizing conventional models is time-consuming and they lack generalization. On the other hand, deep learning models made significant progress by automatically generating relevant features in the latent space to learn underlying patterns of activities [15]. Over the past few years, thanks to heterogeneous publicly available HAR datasets, scholars have implemented various models to recognize human actions. Although these models could achieve high accuracy, one limitation of such studies is that they are not validated against a real case scenario or unseen data. Consequently, model generalization is still a challenge [15,16].

Among deep learning architectures, auto-encoders as an unsupervised deep learning model are suggested for automatic feature extraction [18,19] and dimensionality reduction [20]. Auto-encoders are unsupervised methods that replicate the input in the output, and consist of an encoder, latent space, and decoder. The encoder compresses the input into a latent space and the decoder reproduces the input. The latent space is a compressed representation of data where the data points shape groups that are related to class labels that are, ideally, useful for classification tasks. The difference between the input and output of an auto-encoder is defined as the reconstruction error, where a lower error on the test set indicates good generalization [18]. Garcia et al. [21] proposed an ensemble of auto-encoders where the key feature of the classification model is reconstruction error. They dedicated one auto-encoder per activity, assuming that an auto-encoder trained on one activity minimizes the reconstruction error on samples with the same label. Therefore, the model passes the new data to all auto-encoders and assigns a label based on the lowest reconstruction error. Moyes et al. [22] highlighted the capability of the multi-channel auto-encoder model in producing domain-invariant feature representation. Their results showed that a multi-channel auto-encoder enables generalization to unseen data. In the other study [23], the authors proposed a multi-channel auto-encoder to learn time series data. They achieved a higher generalization using such a multi-channel architecture for anomaly detection.

Multi-task learning is a machine learning approach to learning several related tasks simultaneously, which helps generalization through shared factors across tasks [18,24]. One example of multi-task learning is a supervised auto-encoder in which the latent space of the auto-encoder is connected to a supervised model [25]. The authors in [25] concluded that supervised auto-encoders improve the model performance, although they did not examine any human activity dataset. Additionally, it has not been carefully investigated and reported in the HAR literature how well an auto-encoder performs when reconstructing IMU signals. Through this study, we discuss the role of signal reconstruction in generating relevant features for activity recognition tasks. Subsequently, if the latent space, exploited by compressing signals, is suitable for IMU signal recreation, it contains informative patterns of the input. Therefore, signal reconstruction is the task that should be learned together with the HAR task by the multi-task learning model. Thus, we will demonstrate the latent features’ shape in the case of a precise signal reconstruction to answer these questions:

- Does such a latent space exist where human activities are recognizable using features generated in an automatic and unsupervised way, due to signal compression and reconstruction?
- How does a classifier affect the latent space created by the unsupervised block in a multi-task learning model?

Although activity classification is the primary goal of HAR, we will treat IMU signal reconstruction the same as the main task to ensure that the model recreates the input signals precisely. We hypothesize that an auto-encoder that can reconstruct the input signals to a reasonable degree shapes a latent space where labels are well represented. We propose a multi-channel asymmetric auto-encoder to compress IMU signals and accurately reconstruct them to examine the quality of extracted features in the latent space for the HAR task. Moreover, we examine the effect of several activation functions on the signal reconstruction. Additionally, we connect a classification head to the auto-encoder’s latent space to create a multi-task learning model, and examine the trade-off between the encoder, decoder, and classifier. We aim to address the generalization problem of HAR models in dealing with unseen data using the multi-task learning model capable of accurate signal reconstruction. We report the state-of-the-art results on the UCI-HAR, MHealth, PAMAP2, and USC-HAD datasets. Also, we validate our model against a wild dataset of alpine skiing activities where skiers recorded data on their smartphones.

We investigate the role of signal reconstruction in representation learning for HAR tasks to increase model generalization in coping with unseen data. To this end, we utilize an auto-encoder-based multi-task learning model to compress input signals in a latent space, reconstruct the input precisely, and classify human activities based on the generated features due to signal reconstruction. We demonstrate the viability of the selected model for this task using four public HAR datasets, as well as a novel alpine skiing dataset. The primary contribution of this work to the HAR field is as follows:

- We investigate how signal reconstruction helps form activity-related dense clusters in the latent space of a multi-task learning model.

In addition, the following secondary contributions were achieved:

- We present a multi-channel, asymmetric auto-encoder to automatically learn features from wearable sensor data and precisely reconstruct input signals.
- We propose a multi-task learning model based on the multi-channel asymmetric auto-encoder architecture to benefit from the robust representation learning due to signal reconstruction and boost model generalization for the HAR task.
- We evaluate the multi-task learning model’s efficacy using four public datasets and compare it to the state of the art.
- We investigate the effect of different activation functions on signal reconstruction and provide a comparison.

The rest of the paper is structured as follows: in the Section 2, we will review the literature and related works. In the Section 3, we introduce the architecture of the multi-channel asymmetric auto-encoder and the multi-task learning model. We also provide the details of the experiment and validation. In the Section 4, we test the model and demonstrate the results in detail. Finally, in the Section 5, we discuss the model and results and provide a conclusion.

## 2. Related Work

Multi-task learning aims to learn several tasks concurrently to improve generalization performance [24]. One approach to MTL is representation learning, which assumes that training data in various learning tasks improves representation learning, boosts the model [26], and decreases over-fitting [24]. Ruder in [27] provides a guideline to select such an auxiliary task to enhance model generalization through shared representations. MTL has been broadly applied to computer vision, bioinformatics, health informatics, federated learning, natural language processing, speech recognition, and so on [24,26,28]. Subsequently, an overview of MTL in the field of HAR is given; however, due to the amount of published research, an exhaustive list is out of scope here—see, e.g., work by Chen et al. [15] for further reading.

In [29], the authors proposed a deep multi-task learning method to solve simple and complex activity recognition tasks jointly. They employ CNN to recognize simple activities and add LSTM layers on top of CNN to solve complex activity recognition tasks. The offered model, called AROMA, benefits from the shared CNN to improve generalization. Saeed et al. [30] highlighted the importance of unsupervised learning exploiting vast amounts of unlabeled data and proposed a self-supervised technique for representation learning from unlabeled sensory data. They demonstrated that the self-supervised technique enables the convolutional model to learn suitable features for the HAR task. Chen et al. in [31] proposed a multi-tasking approach to simultaneously address activity recognition (AR) and user recognition (UR) tasks using wearable sensors. They utilized a mutual attention mechanism to enable knowledge sharing between AR and UR tasks.

### 2.1. Use of Auto-Encoders for MTL

Specifically in the frame of HAR and MTL, auto-encoders have found much use, due to their inherent capability of constructing latent spaces that compress time-domain signals which are typical for HAR applications. Auto-encoders were found to be applicable to data derived from various sensor technologies, such as smartphones [32,33,34,35], radar [36], cameras [37], IoT devices [38], and smart homes [39].

In [40], the authors expanded auto-encoders to multi-task learning to recognize concurrent human activities. They defined HAR as a set prediction problem where every set can contain multiple human actions. In their study, a symmetric auto-encoder, inspired by [41], is employed as the unsupervised feature extractor. A classifier is also developed on top of the auto-encoder to learn simultaneously from extracted features and target sets of activities. Similarly, Ma et al. [42] proposed a deep clustering model for HAR applications that relies on an auto-encoder for feature learning. They offered a multi-task learning model to learn three tasks of feature extraction, clustering, and classification concurrently. In another study, Suh et al. [43] proposed a multi-task learning framework that adopts an adversarial encoder–decoder with the maximum mean discrepancy regularization to improve subject generalization.

Similar to the design approach in [40,42,43], this work connects the HAR task head to the compressed, latent space, thus utilizing the same general approach. However, none of the outlined works investigate whether the auto-encoder can recreate the input precisely in the output, instead reporting only on the classification task. It is thus unclear how the quality of the reconstructed signal affects the representation learning and classification task.

### 2.2. State of the Art in HAR

The subsequent works represent the current state of the art in HAR and are used as a reference for comparisons in Section 4.3. Abbaspour et al. [44] examined the role of a combination of Convolutional Neural Networks (CNNs) and Recurrent Neural Networks (RNNs) in HAR. They integrated CNN with four RNNs, i.e., LSTMs, BiLSTMs, GRUs, and BiGRUs, and tested these models on the PAMAP2 dataset. The authors of [45] argued the disadvantage of hybrid models in learning spatio-temporal context from the latent space. Thus, they proposed a self-attention-based neural network model to address this issue by generating a high-dimensional feature space. They validated their method on PAMAP2, Opportunity, Skoda, and USC-HAD. Gao et al. [46] mentioned the difficulties of RNNs in feature representation and offered a dual attention method for HAR. They added channel and temporal attention heads to CNNs and tested their model on PAMAP2 and the other datasets. Abedin et al. [47] offered a cross-channel interaction encoder, including a self-attention mechanism followed by an attentional GRU encoder to improve representation learning by extracting latent relationships between sensor channels. They reported their model’s efficacy on four datasets including PAMAP2.

The authors of [48] proposed a multi-scale Deep Convolution Neural Network (DCNN) ensemble to extract multiple temporal scale features on each sensor separately. They validated their approach against seven publicly available datasets, such as MHealth and USC-HAD. The authors of [49] focused on capturing the spatial and temporal features. They designed a residual block followed by a bi-directional LSTM to capture relevant features for HAR. In [50], the authors proposed a hybrid model to improve the HAR task accuracy and reduce the number of parameters. They designed a new model containing Bi-GRU layers and an inception block to extract temporal and spatial features. Ek et al. [51] presented the Human Activity Recognition Transformer (HART) model for mobile devices, which benefits from a vision transformer for image recognition [52]. They tried to address the heavyweight problem of transformer-based models by proposing a lightweight architecture for mobile devices. Zhang et al., in [53], suggest combining CNN, residual BiLSTM (ResBLSTM), and attention mechanism to cope with similar human actions. They reported an F1 score higher than 98% on the UCI-HAR dataset. The authors of [54] proposed a ConvTransformer model that blends CNN, transformer, and attention mechanism to improve representation learning by extracting global temporal and local spatial features. They tested their model using four public datasets, including PAMAP2 and USC-HAD.

In the literature on alpine skiing, activity recognition using wearables is limited [55]. Scholars usually considered different bio-mechanical aspects of skiing rather than activity recognition while activity recognition is necessary to know the mechanism of injury [56]. The authors of [57] classified alpine skiing styles using a global navigation satellite system (GNSS) and IMU. They extracted domain-related features from advanced or expert skiers to address the HAR task, and classified alpine skiing styles into parallel (drifted or carved) and non-parallel (snowplow or snowplow-steering) turns. In our examination, we considered four parallel skiing styles, which are categorized at the third level of the teaching plan, from start to perfection in four stages, by a national skiing association, and observed skiers with different skill levels.

## 3. Approach and Methods

**Figure 1:**
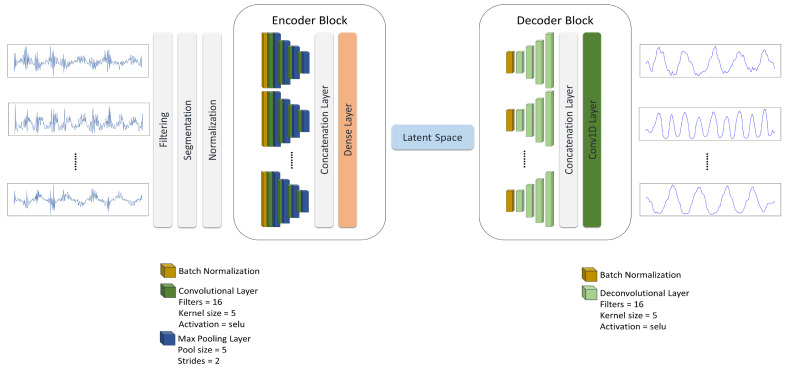
The multi-channel asymmetric auto-encoder. The multi-channel asymmetric auto-encoder is composed of an encoder block that dedicates one encoder to each sensor channel and a decoder block to reconstruct every signal in the output. Every encoder starts with a batch normalization layer followed by four repetitions of the convolutional layer and max pooling layer. Each decoder is made up of a batch normalization layer followed by four deconvolutional layers. All of the convolutional and deconvolutional layers share the same features. The max pooling layer is utilized for down-sampling. It is also used to examine the impact of signal reconstruction on representation learning as the unsupervised baseline.

**Figure 2:**
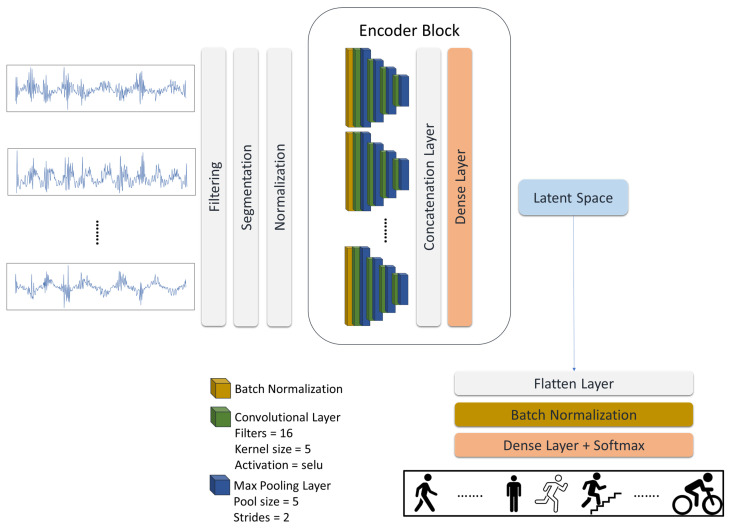
The supervised baseline. Inspired by the unsupervised multi-channel asymmetric auto-encoder, this supervised baseline only uses the encoder block to perform activity classification. It is also used to examine the recognition performance in the absence of signal reconstruction.

**Figure 3:**
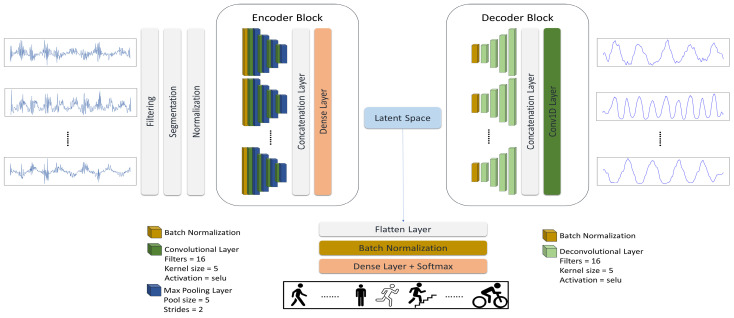
The proposed multi-task learning model (MCAE). The proposed model architecture combines the unsupervised (signal reconstruction) and supervised (HAR classification) tasks. Thus, this multi-task learning model is formed of the multi-channel asymmetric auto-encoder and the classification head for the HAR task. The classification head flattens the latent features and passes them to a batch normalization layer followed by a dense layer with Softmax activation function.

To test our hypothesis and answer the proposed questions, we present a multi-task learning model consisting of a multi-channel asymmetric auto-encoder to reconstruct IMU signals and a classification head to execute the HAR task. First, we explore signal reconstruction using the proposed auto-encoder as the unsupervised baseline introduced in Figure 1 and plot its latent space to see how it shapes the latent features. Second, we examine the HAR classification task as the supervised baseline presented in Figure 2 to compare supervised and unsupervised latent representations. Further, we study how the unsupervised and supervised baseline of the model affect the latent features when they work together in a multi-task learning architecture, introduced in Figure 3.

### 3.1. Model Architecture

We propose a novel multi-channel asymmetric auto-encoder to compress IMU signals, create a latent representation, learn temporal features, and reconstruct the input, Figure 1. The auto-encoder consists of an encoder block and a decoder block. The encoder block dedicates one encoder to each sensor channel to compress the associated signal. At the end of the encoder block, one dense layer fuses all the encoded signals to shape the latent space. Conversely, one decoder recreates every input from the latent features. The last layer of the decoder block is one convolutional layer to aggregate decoded signals.

In the encoder block, every input channel passes through a dedicated encoder composed of a batch normalization layer followed by four repetitions of a convolutional layer, each paired with a down-sampling layer. On the other hand, every decoder in the decoder block up-samples the compressed data via a batch normalization layer followed by four deconvolutional layers. We examine the effect of several activation functions in reconstructing human activity signals recorded by wearables.

Additionally, we removed the decoder and attached a classification head to create a supervised baseline to classify human activities, Figure 2. The classification head is made up of a flatten layer followed by a batch normalization and a dense layer with a Softmax activation function directly connected to the latent space.

Lastly, we introduce the multi-task learning model (MCAE), which combines the multi-channel asymmetric auto-encoder and a classification head, Figure 3. We keep the classifier head as simple as possible to examine the goodness of generated features in the latent space, assuming that a representative latent space does not need a sophisticated classification algorithm. Figure 3 provides the big picture of the model architecture.

The multi-task learning pipeline works as follows: first, the recorded signals go through the preprocessing stage, including filtering, segmentation, and standardization to form the input. In the data preparation phase, we filter the data following the approach in [58]. Then, the smooth signals are segmented by a windowing strategy. After, the pipeline normalizes the data to remove the mean of signals using Standardscaler [59]. The normalized data are passed directly to the model. Then, the encoder block creates a shared latent space for the decoder and classifier. Accordingly, our multi-tasking model has two outputs: the signal reconstruction and the HAR task.

As loss functions for the signal reconstruction and the classification tasks, Huber loss and cross-entropy were chosen, respectively, which are used for robust regression [60] and classification [61]. We used the Adam optimizer with a learning rate of 0.001 and the default hyperparameter and set the batch size to 128 for all the analyses. We implemented our experiment using Tensorflow and Keras and ran the model on Intel(R) Core(TM) i7-7820HQ CPU @ 2.90 GHz 2.90 GHz, Nvidia Quadro M2200, and 32 GB of installed RAM. We set an early stop to stop training when the validation loss stops improving by a minimum change of 0.0001 after 10 epochs.

### 3.2. Datasets

We will test our approach against five data sets, of which one is recorded in the wild. In this study, we analyze four public datasets, UCI-HAR [58], mHealth [62], PAMAP2 [63], and USC-HAD [64]. These public datasets have been widely used in the HAR literature [65]. The fifth dataset is an alpine skiing dataset [66].

**UCI-HAR dataset** [58] consists of six activities of daily life such as walking, walking upstairs, walking downstairs, sitting, standing, and lying. A group of thirty subjects performed these activities while wearing a smartphone on the waist. For each session, 3D linear acceleration and 3D angular velocity with a sampling rate of 50 Hz are recorded. The UCI-HAR dataset is already segmented, preprocessed, and divided into two sets of 21 subjects for training (70%) and 9 subjects for testing (30% unseen subjects). The dataset is filtered and segmented by a window size of 128 samples and a 50% sliding rate.

**The MHEALTH (Mobile HEALTH) dataset** [62] contains acceleration, angular velocity, magnetic field orientation, and ECG measurements from ten subjects while performing several physical activities, such as cycling, jogging, running, waist bends forward, etc. The sensors on the subject’s chest, right wrist, and left ankle measure each user’s motion. The sensor on the chest provides 2-lead ECG measurements. All the signals are sampled at a frequency of 50 Hz. For the validation, we reserve three subjects, roughly 30% of the dataset. The dataset contains twelve activities of daily life and sports. Subjects performed the activities at their best in the wild. The complete description of data collection is in the paper [62].

**The PAMAP2 Physical Activity Monitoring dataset** [63] provides motion signals of twelve different physical activities, for instance, walking, running, cycling, and Nordic walking, collected from nine subjects, including one female. The dataset includes IMU signals from the sensors attached to the wrist, chest, and ankle recorded at a 100 Hz sampling rate. It also provides a heart rate signal recorded at a 9 Hz frequency. For validation, we reserve two objects.

**USC-HAD dataset** [64] consists of twelve low-level activities where some of the activities are very similar, such as walking forward, walking left, walking right, walking upstairs, and walking downstairs. A group of fourteen volunteers performed the activities while wearing a sensor at their front right hip. The acceleration and angular velocity signals were collected at a sampling rate of 100 Hz using MotionNode.

**Alpine skiing dataset** is composed of four different skiing styles, Parallel Basic—Long, Parallel Basic—Short, Parallel Dynamic—Long, and Parallel Dynamic—Short, performed by a group of recreational alpine skiers with different skills. The importance of this dataset is that some sessions are recorded uncontrolled which makes it appropriate for a real case scenario test. Please refer to [66] for detailed information. It is worth mentioning that some of the skiers performed skiing activities in different locations.

We summarize some characteristics of these datasets in Table 1.

### 3.3. Validation

In this study, we validate our model against data from unseen users. Therefore, we reserve several subjects from each dataset to create test sets. We do not introduce the unseen data to the model at any stage of training. We use the data from the other subjects for training and validation. Thus, to measure our model generalization performance, we partition our data into three sets, a training set to train the model, a validation set to tune hyperparameters, and a test set to measure the generalization performance [67]. The test set includes unseen users and is independent of the other two sets.

The authors of the UCI-HAR dataset already divided the dataset into two groups of subjects for model training, where they subselected nine random subjects as a test set. These users constitute 30% of the dataset. Similarly, we reserve a number of subjects from each dataset, which amounts to roughly 30% of the whole dataset. From the MHealth dataset, we reserve three random users. To validate our model by PAMAP2, we reserve users five and six, which are chosen for testing in the other studies [49,54]. Among volunteers from the USC-HAD dataset, we reserve four of them, who are selected to test the model in [54]. Among skiers in the alpine skiing dataset, we chose two subjects with different skills and reserved all their sessions. These two users are recreational alpine skiers who recorded their data by themselves in the wild. Therefore, they simulate the real case scenario to some extent.

### 3.4. Evaluation Metrics

To evaluate the accuracy of our proposed model, we produce a classification report on every HAR task which provides accuracy, precision, recall, and F1 score metrics [68]. Additionally, for each task, we provide a confusion matrix to compare classification results and assess the model performance. Moreover, we visualize the latent features to show how effectively the model is at creating useful features.

### 3.5. Data Analysis

First, we examine the unsupervised baseline on the UCI-HAR to see how it reconstructs the signals and provide some examples of signal reconstruction. Second, we plot and compare the generated latent features by unsupervised and supervised baselines on the UCI-HAR dataset. Then, we validate our proposed multi-task learning model on the other datasets to assess the model’s performance in recognizing various activities. Additionally, we examine how a model performs without signal reconstruction as a single-task learning model using the supervised baseline, see Figure 2. In the end, we provide a detailed comparison. Therefore, we not only evaluate our model’s performance via classification metrics but also check how model baselines influence the latent features and, consequently, the overall performance.

## 4. Results

In this section, we first illustrate the result of signal reconstruction and compare the extracted features using unsupervised and supervised baselines. Then, we present the result of the multi-task learning model on the HAR task. Finally, we provide a comparison of our approach with the state of the art.

### 4.1. Signal Reconstruction

In this study, we are exploring how an accurate signal reconstruction using auto-encoders shapes the latent space. Indeed, we try to answer the following question:

- Does such a latent space exist where human activities are recognizable using features generated in an automatic and unsupervised way, due to signal compression and reconstruction?

**Figure 4:**
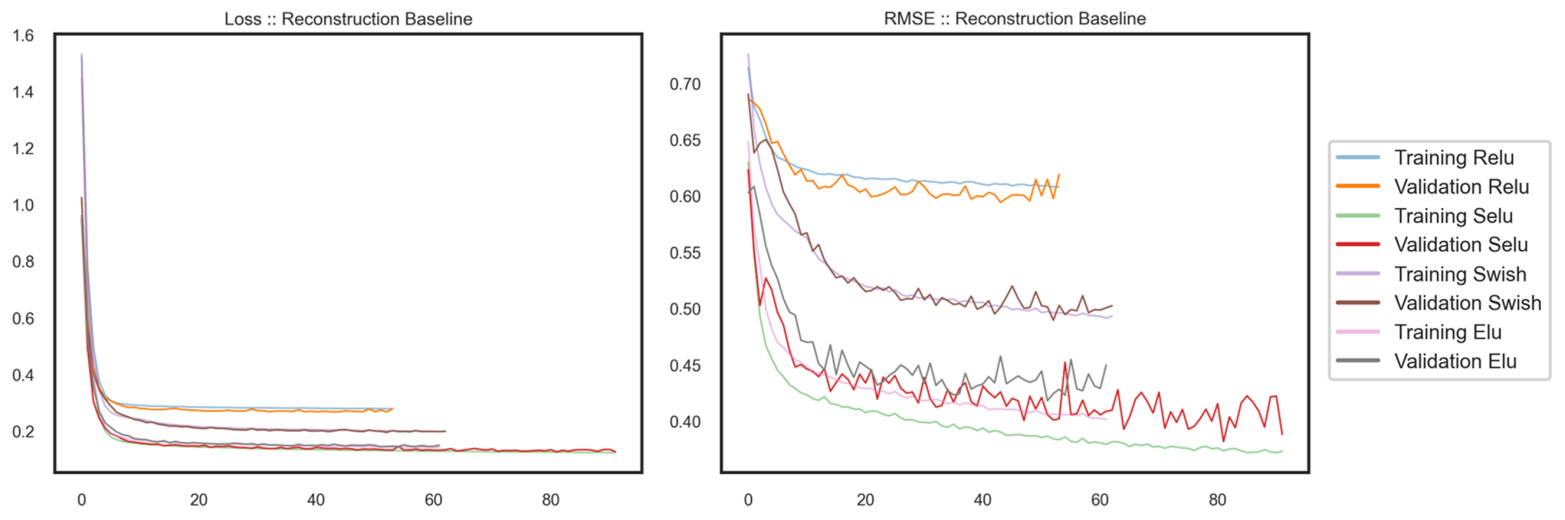
The loss and Root Mean Square Error (RMSE) of the unsupervised baseline for signal reconstruction using different activation functions on the UCI-HAR dataset. Although all the loss values look similar, Selu performs better, which is represented when looking at RMSE.

Therefore, we examine the unsupervised baseline to see how automatically generated features form the latent space. The expectation is that even similar activities are far from each other in the latent space. We investigate the role of different activation functions in reconstructing IMU signals. Our preliminary results on signal reconstruction showed that Relu [69] has tribulations with reproducing non-linear periodic signals. Therefore, we investigate other activation functions such as Selu [70], Elu [71], and Swish [72] in this study to have a better comparison. The signal reconstruction results on the UCI-HAR dataset show that all activation functions have similar performance when looking at loss, Figure 4. However, Selu performs better on periodic activities such as walking, which is why it has a better Root Mean Square Error (RMSE), Figure 4. Relu, Elu, and Swish mostly converge to the mean, a valid reconstruction for inactive activities such as sitting. Therefore, we only used Selu in our architecture.

**Figure 5:**
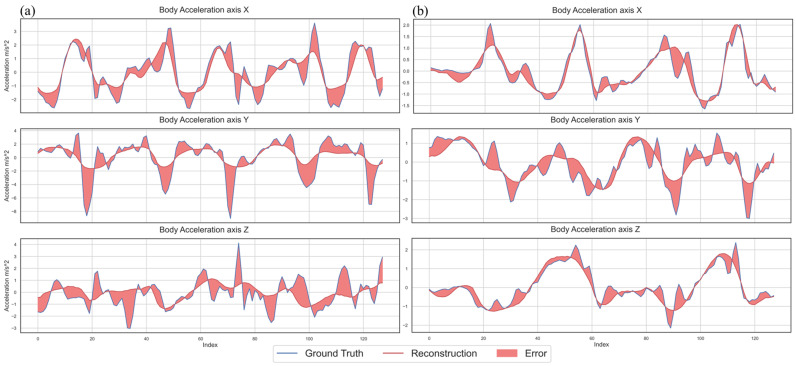
Signal reconstruction from the UCI-HAR dataset using the unsupervised baseline and Selu activation function. (**a**) shows the worst signal reconstruction in the dataset regarding RMSE which is from walking activity. On the other hand, (**b**) presents the best reconstruction of walking activity as a comparison.

We wish to examine whether the proposed multi-channel asymmetric auto-encoder can recreate the input in the output and demonstrate how signal reconstruction helps form activity-related dense clusters in the latent space of the model. Figure 5 shows two examples of signal reconstruction using the Selu activation function where (a) illustrates the worst reconstruction result, which belongs to the walking class label. On the other hand, (b) demonstrates the best reconstruction of walking samples. As Figure 5 implies, the model can reproduce a smooth pattern of activities in the output even in the worst-case scenario.

**Figure 6:**
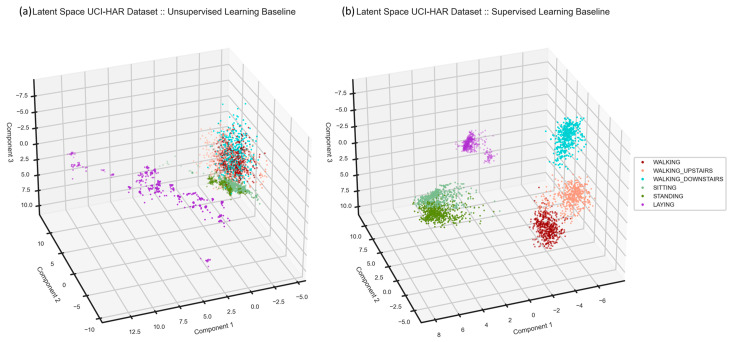
Latent space comparison. As one sees, in (**a**), the latent space of the unsupervised baseline, one of the activities, lying, is far from the others. The other activities are closer to each other. They shape two clusters, though. We can see that walking activities shape one cluster on the top. Additionally, sitting and standing form another cluster in the middle. On the other hand, in the latent space of the supervised baseline, (**b**), activities are well separated. However, sitting and standing are pretty close, and there is a negligible overlap between walking and walking upstairs.

The next step is to see if generated features in the latent space represent any difference between class labels. We applied principal component analysis (PCA) on the feature space and plotted three components in Figure 6. We use PCA only to aid the visualization. One can see that one of the activities, lying, is separated from the others while the other activities are close together and shape two groups. Although the model reconstructs input signals adequately, its latent space seems not representative at first glance. However, the model creates three clusters, including similar activities of walking, sitting and standing, and lying. In contrast to the unsupervised model, the supervised baseline creates a more representative feature space of activities. However, sitting and standing are well separated in the latent space. Even though the supervised baseline performs very well in the HAR task, we will examine the cooperation of these two baselines in the multi-task learning architecture in the next section. At the end of the results section, we compare the performance of the proposed multi-task learning with its supervised baseline.

The outcome of the first phase of our analysis suggests that a signal reconstruction model alone cannot create a representative feature space.

### 4.2. Multi-Task Learning

For the sake of an answer to the first question, we examined the feature representation in the latent space. Although the unsupervised model could create some groups of similar activities, we could not conclude that a signal reconstruction alone creates a representative feature space. Therefore, we expand the unsupervised model by adding a classification to its latent space to check whether it learns from latent features and recognizes activities. This expansion helps us to answer the second proposed question: how does a classifier affect the latent space created by the unsupervised block in a multi-task learning model? We expect the trade-off between the decoder and classifier to reform the latent space. We examine the effect of the classifier on the latent space to provide an answer to the second question and test the model’s performance using four public datasets and state-of-the-art results on them.

#### 4.2.1. UCI-HAR

**Figure 7:**
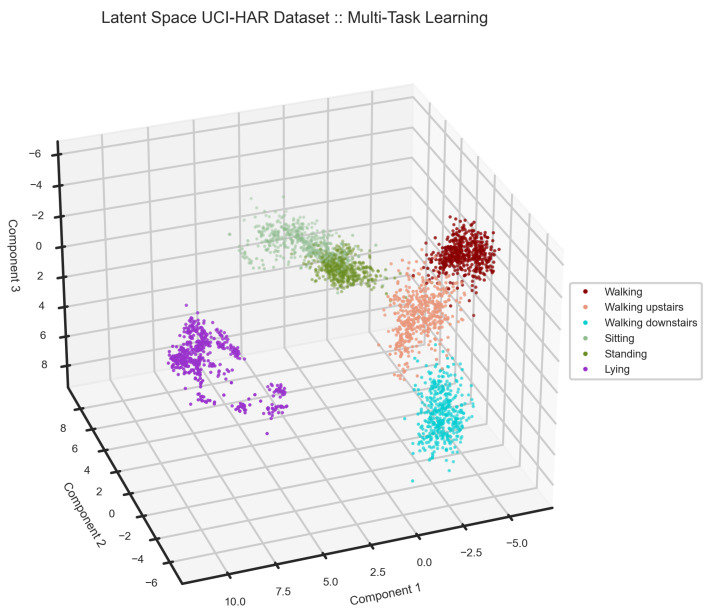
In the latent space of the UCI-HAR dataset, activities are separable. There is only a small overlap between sitting and standing.

**Figure 8:**
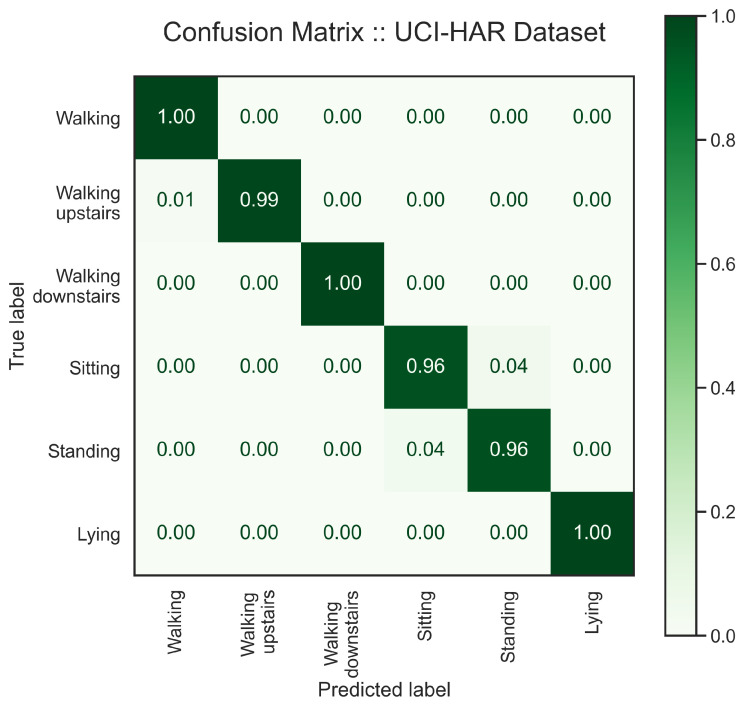
The confusion matrix shows the model’s efficacy in recognizing activities in the UCI-HAR dataset with a high accuracy. As one can see, the overlap between sitting and standing in Figure 7 is well represented in the matrix where the accuracy drops to 96%.

We present the test result on the UCI-HAR dataset in Table 2, and Figure 7 and Figure 8. As anticipated, the latent space changed in favor of the classification task. Figure 7 illustrates activities in the latent space, where they are far from each other in comparison with the latent representation extracted by the unsupervised model in Figure 6. This separation between activities is well represented in the confusion matrix, Figure 8, where there is no confusion between the clusters of lying, sitting and standing, and walking activities. As a result, the model benefited from such representation learning and performed remarkably well in activity recognition with an F1 score of 99%. However, there is still a little confusion between sitting and standing. We provide a classification report in Table 2 to elaborate on the results and support our claims.

#### 4.2.2. MHealth

**Figure 9:**
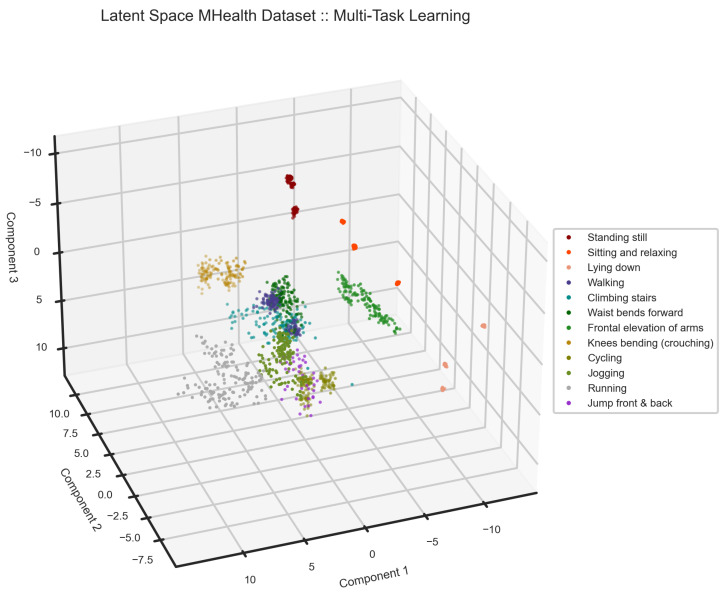
In the latent space of the MHealth dataset, static activities, such as standing and lying, are far from the others. Activities performed in a fixed position such as the frontal elevation of arms and knee bending are well separated from the other activities.

**Figure 10:**
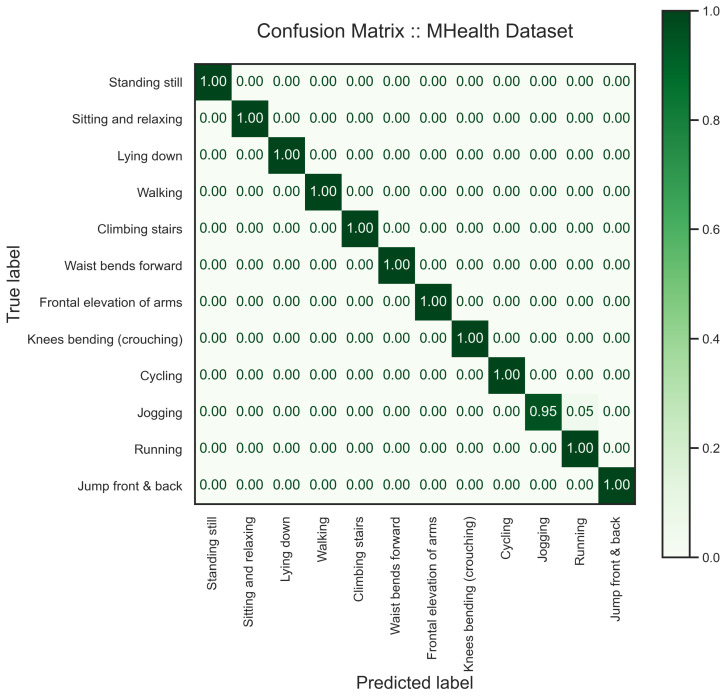
The confusion matrix shows the performance of the proposed multi-task learning model on the MHealth dataset. We chose three random subjects for the test set who are independent of the training set.

We segmented the MHealth dataset using a window size of 2.56 s and a sliding rate of 50%. Three subjects were randomly chosen to test the model. The samples from three subjects are roughly 30% of the whole dataset. We present the performance of our proposed model on the mHealth dataset in Table 3, Figure 9 and Figure 10. The model performed accurately in the detection of various activities from unseen subjects, except there is a confusion between Running and Jogging that can also be seen in the latent features, Figure 9. In the latent space of the model, one can see that generated features on MHealth data are well separated. As one can see, static activities and activities in fixed positions, i.e., sitting and relaxing and frontal elevation of arms, are far from other activities, such as walking or cycling.

#### 4.2.3. PAMAP2

**Figure 11:**
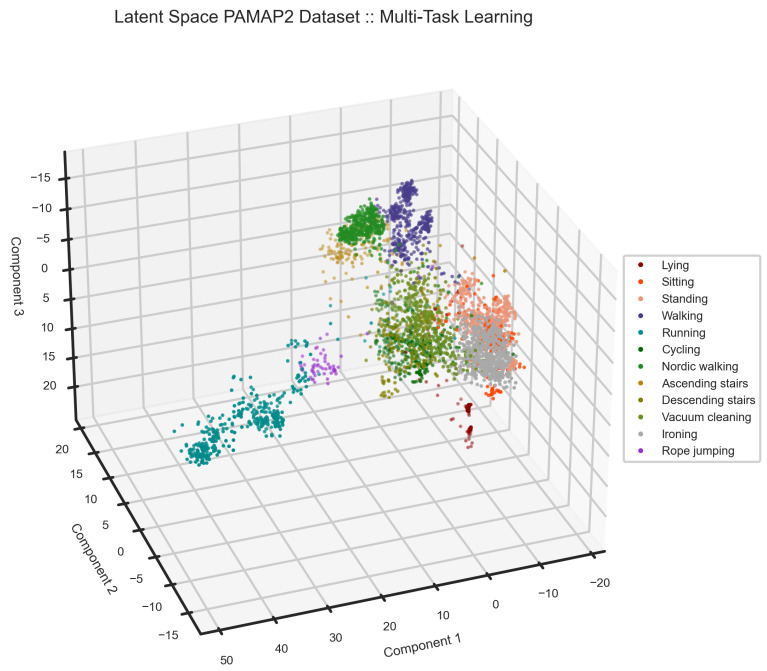
The latent space of the model on the PAMAP2 dataset can clarify why the model performance is lower in comparison to the MHealth and UCI-HAR datasets. On the right side of the latent space, sitting, standing, and ironing shape one cluster. This is where, dissimilar to the other datasets, our model has a problem in separating static activities. This could be due to the fact that standing and ironing share similar patterns on the sensors attached to the ankle and chest.

**Figure 12:**
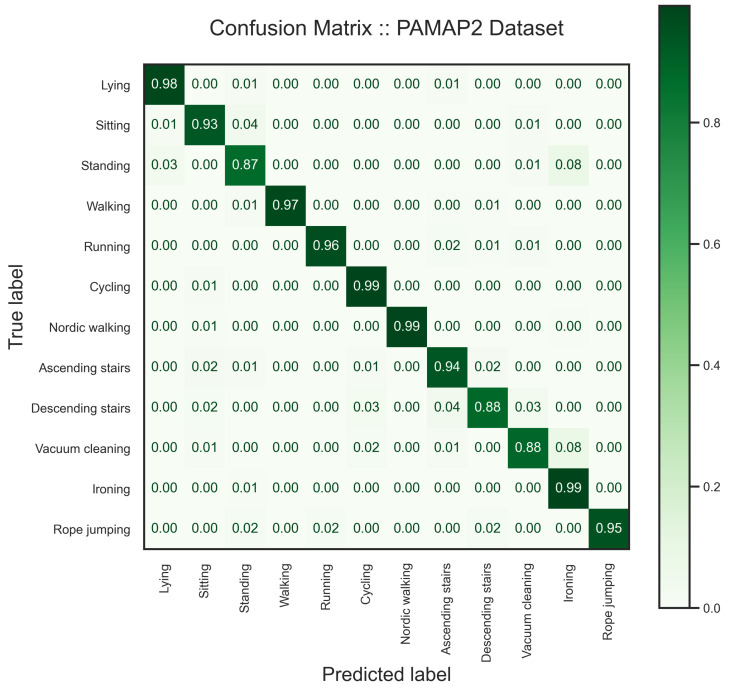
As the confusion matrix of the trained model on the PAMAP2 dataset suggests, the model performs well in the HAR task except where it struggles in distinguishing vacuum cleaning and standing from ironing.

The results of the analysis on the PAMAP2 dataset are presented below, employing a window size of 5.12 s with a 25% sliding rate to capture temporal dynamics effectively. To assess the robustness of our model, we selected two subjects for performance evaluation, representing approximately 30% of the entire dataset. The experimental results, as shown in Table 4, Figure 11 and Figure 12, reveal a decrease in the F1 score, particularly in the recognition of the standing activity. This decline is attributed to the model’s challenge in distinguishing between various static activities, such as standing, vacuum cleaning, and ironing. Additionally, the model exhibits confusion in discriminating between ascending and descending stairs. Figure 11 visually illustrates the separation of activities within the latent space, showcasing distinct clusters for each activity. The observed overlap in the latent space provides insights into the model’s performance, particularly in explaining its difficulty in accurately differentiating static activities. Notably, our model has challenges distinguishing the static activities of this dataset compared with the other datasets.

#### 4.2.4. USC-HAD

**Figure 13:**
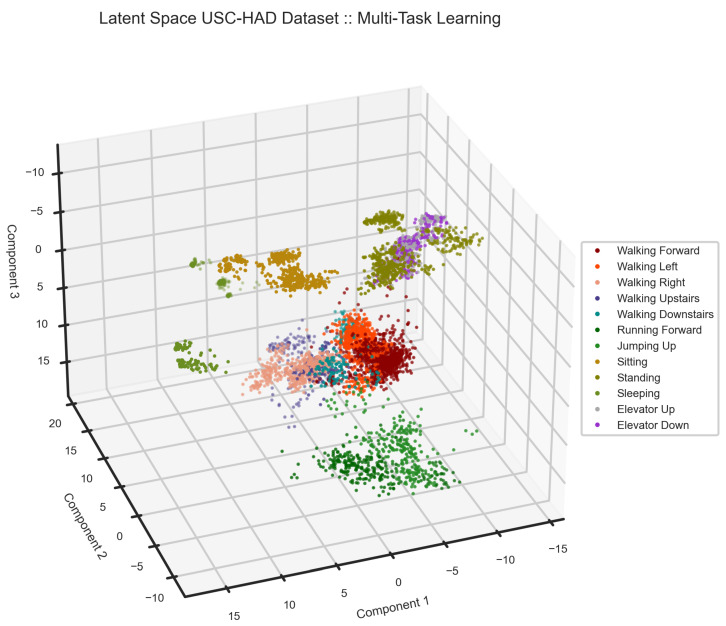
The latent space of the multi-task learning model on the USC-HAD dataset. As one can see on the top right of the scatter plot, the two activities of elevator up and down are not distinctive. Additionally, they share the space with standing.

**Figure 14:**
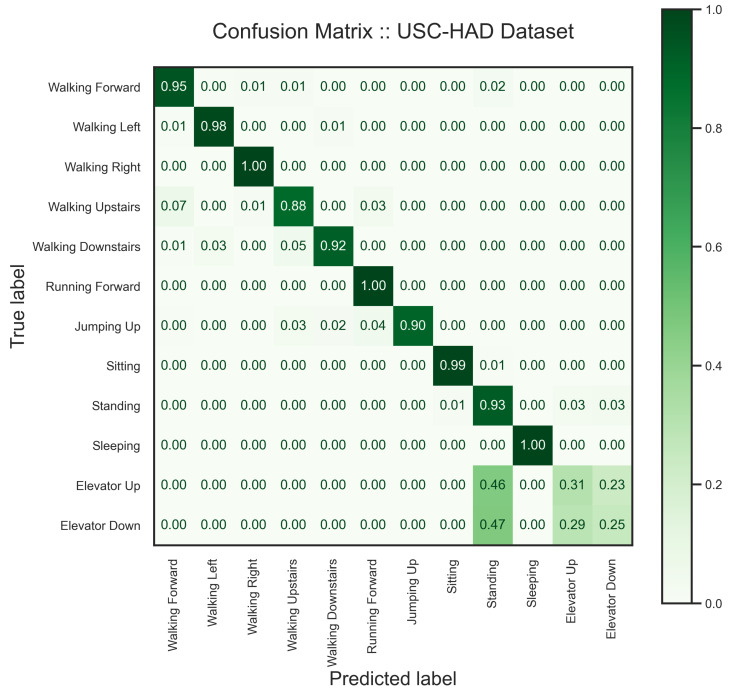
Confusion matrix of the trained model on the USC-HAD dataset. The accuracy drop in the classification report is well represented in the confusion matrix where two activities, elevator up and elevator down, are classified as standing.

To analyze the USC-HAD dataset [64], we chose a window size of 5 s with 75% overlap to segment the data. Then, we reserved four users to validate our model. The classification report, Table 5, the latent space, Figure 13, and confusion matrix, Figure 14 provide detailed information on the model performance on the USC-HAD dataset. As one can see in the classification results, the accuracy on this dataset dropped drastically, as there are various similar activities. However, the model performance is of the highest in the literature [48,73]. The main challenge of this dataset is similar activities. The major group of indistinguishable activities is standing, standing in an elevator going up, and standing in an elevator going down. Additionally, five variations of walking challenge the representation learning and classification task. The latent space of the model is depicted in Figure 13.

#### 4.2.5. Alpine Skiing

**Figure 15:**
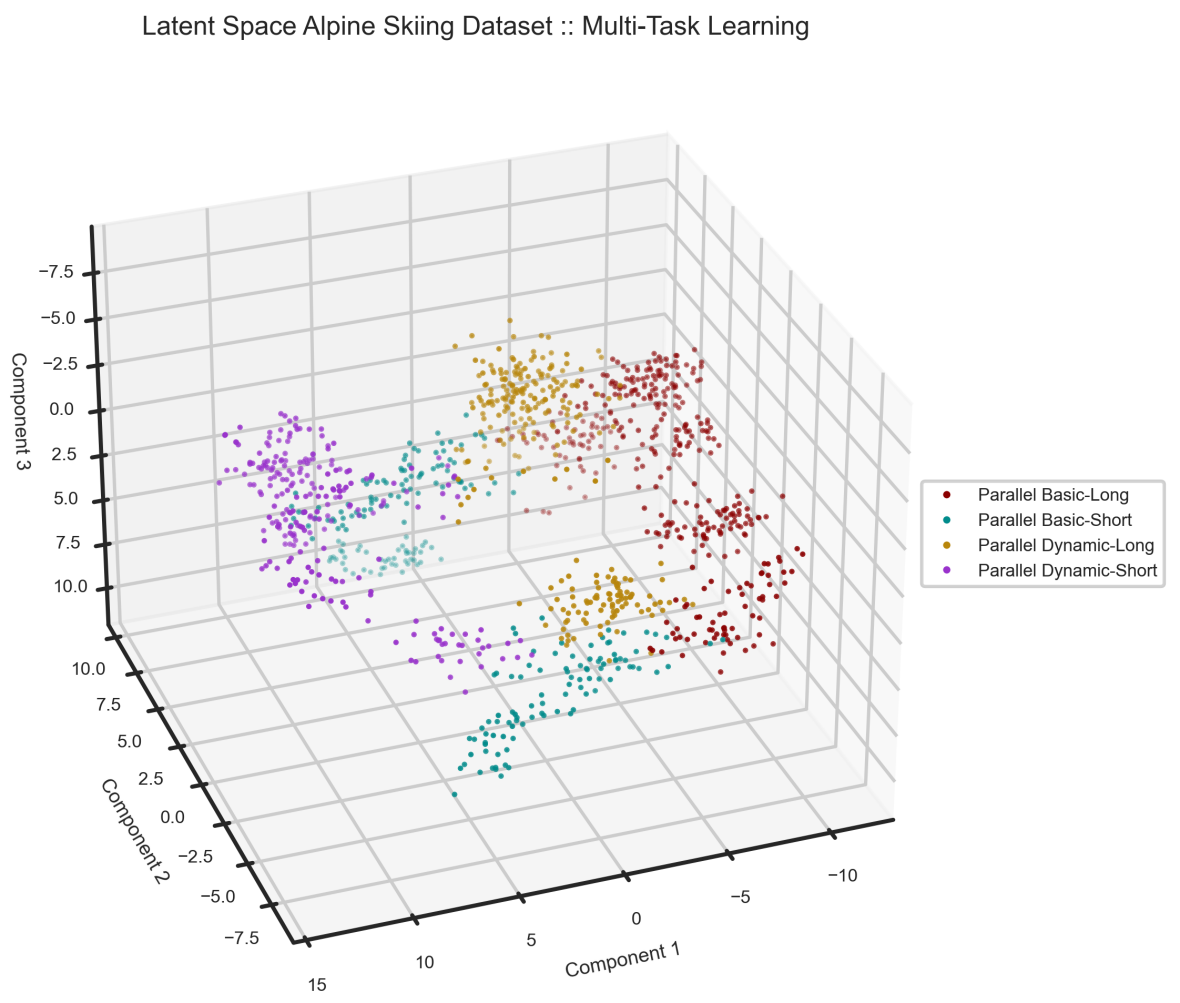
The latent space of multi-task learning model on the alpine skiing dataset. As one can see, Parallel Basic—Long and Parallel Dynamic—Short are far from each other as an indication of why there is no confusion between these two techniques in the confusion matrix.

**Figure 16:**
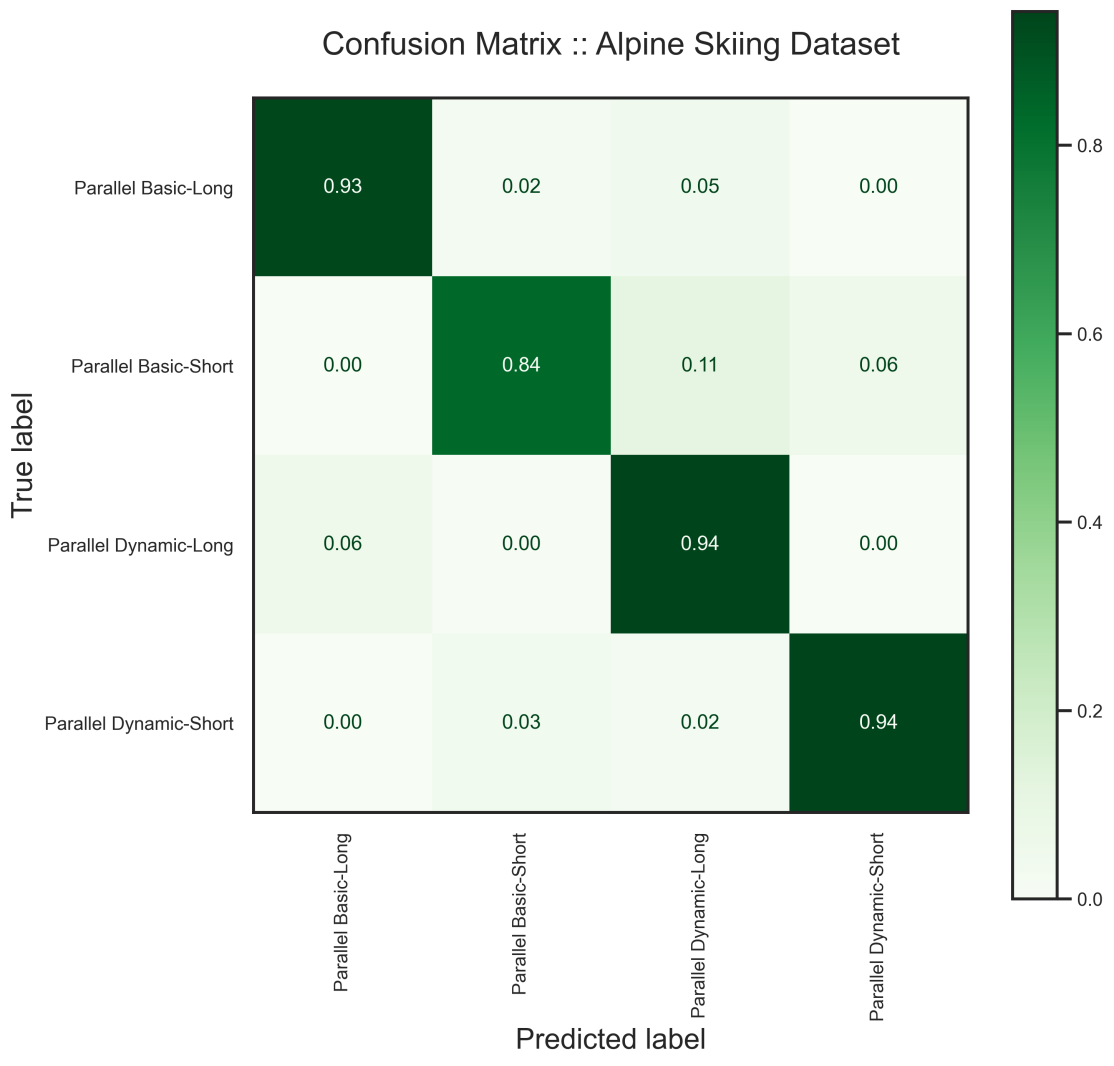
The confusion matrix of the proposed model on the alpine skiing dataset shows that the model has some difficulty in distinguishing Parallel Dynamic—Short from the other activities. As it is represented in the latent space, there is no confusion between Parallel Basic—Long and Parallel Dynamic—Short.

We segmented the data into windows of 10 s with a 25% sliding rate. From all the recordings, we subselected all the trials performed by two of the skiers with separate skill levels for testing the model. As explained in [66], skiers with different levels of expertise produce different patterns, which makes the HAR task more challenging. One of the skiers is an experienced recreational skier, considered an expert, and the other subject is an alpine skiing enthusiast, evaluated as a novice. Moreover, users’ data is recorded under various conditions on their own smartphones in an uncontrolled manner. Therefore, this is a demanding task for the proposed model to prove its generalization. Unlike the other use cases, we followed the data preparation suggested in [66] to fix the orientation. The inputs to the model are acceleration in the world reference and magnetic field channels. The results on the alpine skiing dataset are shown in Table 6, Figure 15 and Figure 16.

We plotted the latent space of our model on the alpine skiing dataset in Figure 15. As one can see, there are some overlaps between samples, which could be the source of confusion in the result. Although these techniques are different in terms of the number of turns and speed, they can be performed similarly under varying circumstances, such as skill level.

### 4.3. Comparison

In this section, we compare the performance of the multi-task learning model with its supervised baseline to see how it performs without the signal reconstruction head. Additionally, we implement a multi-task learning model using a classical auto-encoder (CAE). In contrast to our model, The CAE dedicates only one encoder and decoder to all the input signals. Therefore, all the input signals share one encoder and one decoder. Moreover, these results are compared to the state of the art for a better assessment.

In Table 7, one can see the supervised baseline works pretty similar to the proposed model on the UCI-HAR, MHealth, and PAMAP2. However, the classifier performance drops significantly on the other two challenging datasets. The USC-HAD contains various similar activities and the alpine skiing dataset includes users with different skills. As reported by the other studies, the overall accuracy of the trained model on the USC-HAD dataset is relatively low compared to the other public datasets. Moreover, CAE’s performance falls considerably on all datasets, especially the alpine skiing dataset.

Note that, in this comparison, we only considered studies that reserved one or more subjects to test their model. Therefore, we do not compare our studies with leave-one-trial-out or random train–test split approaches. Additionally, some scholars in this comparison have suggested several models; nevertheless, we reported the best performance among those models.

## 5. Discussion

This study aims to investigate the role of signal reconstruction in representation learning to boost model generalization. We proposed a multi-channel asymmetric auto-encoder to reconstruct IMU signals and generate temporal features in the latent space. Initially, we investigated the role of different activation functions in reconstructing IMU signals. Further, we added a classification head to the unsupervised model to offer a multi-task learning architecture for HAR. Finally, we tested our model using four publicly available datasets and an alpine skiing dataset.

We proposed a novel multi-channel asymmetric auto-encoder to reconstruct the IMU signals and examined how signal reconstruction improves model generalization for the HAR task. Our quantitative (Figure 4) and qualitative results (Figure 5) show that the right choice of activation function reduces the reconstruction error and can help recreate IMU signals. Further, we formed a latent representation using the unsupervised baseline capable of precise signal reconstruction to answer the first proposed question: does such a latent space exist where human activities are recognizable using features generated in an automatic and unsupervised way, due to signal compression and reconstruction? Although the shaped latent space on UCI-HAR (Figure 6) was not perfectly representative of the activities, the unsupervised model created several groups of similar activities. Thus, we concluded that a precise signal reconstruction alone cannot form a latent space where all the activities are recognizable.

Further, we added a classification head to the multi-channel asymmetric auto-encoder to develop a multi-task learning model, which benefits from the model generalization to address the HAR task. Also, we demonstrated “How does a classifier affect the latent space created by the unsupervised block in a multi-task learning model?” in Figure 6 and Figure 7. We examined the HAR task performance against unseen data from various datasets (Table 2, Table 3, Table 4, Table 5 and Table 6). Our results (Figure 7, Figure 8, Figure 9, Figure 10, Figure 11, Figure 12, Figure 13, Figure 14 and Figure 15) show that the signal reconstruction helps the model generalization considerably, especially when activities are complex, such as skiing and crunching, or very similar, such as running and jogging. This can be verified by the outcomes from the supervised baseline, shown in Table 7, which exhibit a decline in accuracy due to the lack of signal reconstruction.

Even though the proposed model achieved high accuracy in the HAR task, it had some difficulties distinguishing two activities, namely “elevator up” and “elevator down”, from standing in the USC-HAD dataset. We believe recognizing such activities requires data recording using other sensors, such as Magnetometer. Additionally, except when an elevator starts moving, there is not much difference between standing anywhere or in the elevator, a confusion we see in Figure 14, as the acceleration is zero when the lift reaches a constant velocity.

Using multi-task learning again raises the following question: “Which tasks should and should not be learned together in one network when employing multi-task learning?” [74]. Although the main purpose of our study is the HAR task, we treated the unsupervised task the same way as the primary goal, to see how these two tasks help the overall performance. In the other studies, regarding what we discussed in the signal reconstruction section, it seems that multi-task learning sacrifices the signal reconstruction task in favor of the HAR task. As “Relu” has been widely used in the literature and showed the poorest performance in signal reconstruction, Figure 4, we investigate the performance of our model using “Relu” activation functions to elaborate on the effect of precise signal reconstruction on the HAR task. We present the results of multi-task learning using different settings in Table 8. Although the performance of the multi-task learning using “Relu” and classical auto-encoder are still acceptable, Table 8, none of these models could precisely reconstruct input signals and often converged to the average of the signal. Consequently, the accuracy of these two models dropped in comparison to the proposed model trained by the “Selu” activation function. Therefore, we argue that the accuracy of auxiliary tasks in multi-task learning impacts the overall performance and should not be sacrificed in favor of the primary task.

The main drawback of the model is perhaps that the number of layers increases with the number of sensors and, consequently, the number of trainable parameters. This issue is more crucial when several sensors are attached to the user’s body. One solution to the problem could be to reshape the input and pass sensors separately, or have all the channels as one input. This resolution reduces the model size dramatically and makes the model very similar to the classical auto-encoder. Although this solution lowers the number of trainable parameters from nearly 90 thousand to about ten thousand on the UCI-HAR dataset, it decreases the model’s accuracy by 4%; as reported in Table 7, the accuracy of MTL is 98.55% while it is 94.61% for CAE.

To keep the size of the proposed model under control, we assigned a lower filter size to the convolutional layers. Among the trained models in our study, the largest is the model trained on the PAMAP2. As a comparison, the lightweight model offered by [50] has about 1.1 million parameters for the trained model on the UCI-HAR dataset, while our model has less than 90 thousand. Additionally, the lightweight model reported by [51] has more than 1.2 million parameters. In Table 9, we provide a short comparison with the other models on the UCI-HAR and PAMAP2 datasets.

Following the suggestion by [65], a robust and generalized model needs no complicated sensor setup and performs well on available sensors. We tested our model on four publicly available datasets with various sensor designs ranging from one to five, including IMU and ECG (Table 1). Our model performed very well and consistently on all these datasets with comparable accuracy to the state of the art (Table 2, Table 3, Table 4, Table 5 and Table 6). Moreover, we tested our model on the alpine skiing dataset, where data from only one IMU sensor is provided to train the model. Regardless of limitations in alpine skiing activities, the model performed very well when we compared our results to a more complicated and inflexible sensor setup, reported by [57] in contrast to the alpine skiing dataset, with no fixed phone orientation and subjects with different skills [66].

## 6. Conclusions and Future Work

This paper presents a multi-task learning model that consists of a classification head to carry out the HAR task and a multi-channel asymmetric auto-encoder to guarantee signal reconstruction. The suggested model makes use of shared representation in both the HAR and signal reconstruction tasks to boost generalization. We compared the results of signal reconstruction using different activation functions and discovered that the “Selu” activation function preserves the periodic activities’ patterns and recreates them in the output. To evaluate the HAR task, we tested the multi-task model performance using public datasets such as UCI-HAR, MHealth, PAMAP2, and USC-HAD. Also, we validated our model via an alpine skiing dataset collected by skiers on their smartphones in the wild. We achieved F1 accuracies of 98.55%, 99.58%, 94.88%, 83.90%, and 91.24% on the UCI-HAR, MHealth, PAMAP2, USC-HAD, and alpine skiing datasets, respectively. Finally, we compared our results with the state of the art. In conclusion, although signal reconstruction alone does not create representative latent features, in combination with the classifier, it generates a robust feature representation. The consistent performance across state-of-the-art datasets shows that the proposed model architecture is robust and generalized.

In this study, we kept the supervised model simple to test the potential of the unsupervised model in representation learning, which can be developed in the future depending on the data and the accuracy requirements, as the model may perform better using a multi-layer classification head. Additionally, we set a lower filter size than usual in the convolutional layers to keep the model size under control. Considering the essentials of the classification task, it might be required to increase the filter size in the convolutional layers. Therefore, there are two options for potential improvements, such as developing a multi-layered classification head and experimenting with the filter size.

Users need a certain level of expertise to perform some activities properly, where the users’ skills can dramatically affect the activities’ pattern. This effect is particularly shown in the alpine skiing dataset when we computed the classification metric for an expert and a novice separately. Our results show that the F1 score drops to 82% for the novice skier, which implies that the recognition task becomes more complicated for skiers with lower skill levels as they introduce more variation in the signal shape. On the other hand, expert skiers perform activities faster and more consistently, which leads to more clear patterns [66]. This difference needs further research to address not only activity recognition but also activity assessment and, as a result, skill level detection in the wild.

## Figures and Tables

**Table 1:** The table represents the number of activities and their types (Activities of Daily Life (ADL) or Sports), the number of sensors and their types, and the number of subjects in each dataset. A: Accelerometer, G: Gyroscope, M: Magnetometer, ECG: Electrocardiogram, HR: Heart Rate.

Dataset	Activities	Activity Type	Sensors	Frequency	Subjects
UCI-HAR	6	ADL	A, G	50 Hz	30
mHealth	12	ADL and Sports	A, G, M, ECG	50 Hz	10
PAMAP2	12	ADL and Sports	A, G, M, HR	100 Hz	9
USC-HAD	12	ADL	A, G	100 Hz	14
Alpine Skiing	4	Sports	A, G, M	50 Hz	8

**Table 2:** Classification report of the proposed multi-task learning model on the UCI-HAR dataset. In this report are F1 score, precision, recall, macro average, and weighted average F1 score. The support column shows the number of samples.

Label	Precision	Recall	F1 Score	Support
walking	0.99	1.00	1.00	496
walking upstairs	1.00	0.99	0.99	471
walking downstairs	1.00	1.00	1.00	420
sitting	0.96	0.96	0.96	491
standing	0.96	0.96	0.96	532
lying	1.00	1.00	1.00	537
accuracy			0.99	2947
macro avg	0.99	0.99	0.99	2947
weighted avg	0.99	0.99	0.99	2947

**Table 3:** Classification report of the proposed multi-task learning model on MHealth dataset.

Label	Precision	Recall	F1 Score	Support
Standing still	1.00	1.00	1.00	141
Sitting and relaxing	1.00	1.0	1.0	141
Lying down	1.00	1.00	1.00	141
Walking	1.00	1.00	1.00	141
Climbing stairs	1.00	1.00	1.00	141
Waist bends forward	1.00	1.00	1.00	137
Frontal elevation of arms	1.00	1.00	1.00	132
Knees bending (crouching)	1.00	1.00	1.00	139
Cycling	1.00	1.00	1.00	141
Jogging	1.00	0.95	0.97	141
Running	0.95	1.00	0.98	141
Jump forward and back	1.00	1.00	1.00	45
Accuracy			0.99	1581
Macro avg	0.99	0.99	0.99	1581
Weighted avg	0.99	0.99	0.99	1581

**Table 4:** Classification report of the proposed multi-task learning model on the PAMAP2 dataset.

Label	Precision	Recall	F1 Score	Support
Lying	0.96	0.98	0.97	357
Sitting	0.95	0.93	0.94	379
Standing	0.91	0.87	0.89	353
Walking	1.00	0.97	0.99	439
Running	1.00	0.96	0.98	360
Cycling	0.96	0.99	097	342
Nordic walking	1.00	0.99	0.99	403
Ascending stairs	0.89	0.94	0.91	206
Descending stairs	0.94	0.88	0.91	179
Vacuum cleaning	0.94	0.88	0.91	346
Ironing	0.90	0.99	0.94	540
Rope jumping	1.00	0.95	0.97	56
Accuracy			0.95	3960
Macro avg	0.95	0.94	0.95	3960
Weighted avg	0.95	0.95	0.95	3960

**Table 5:** Classification report on the USC-HAD shows serious confusion between standing, elevator up, and elevator down.

Label	Precision	Recall	F1 Score	Support
Walking Forward	0.98	0.95	0.96	1004
Walking Left	0.98	0.98	0.98	473
Walking Right	0.97	1.00	0.99	465
Walking Upstairs	0.87	0.88	0.88	235
Walking Downstairs	0.93	0.92	0.92	200
Running Forward	0.94	1.00	0.97	279
Jumping Up	1.00	0.90	0.95	232
Sitting	0.99	0.99	0.99	554
Standing	0.62	0.93	0.74	574
Sleeping	1.00	1.00	1.00	700
Elevator Up	0.47	0.31	0.37	317
Elevator Down	0.47	0.25	0.32	333
Accuracy			0.88	5366
Macro avg	0.85	0.84	0.84	5366
Weighted avg	0.87	0.88	0.87	5366

**Table 6:** Classification report on the alpine skiing dataset shows the highest confusion on the Parallel Basic—Short, where it is classified as Parallel Dynamic—Long.

Label	Precision	Recall	F1 Score	Support
Parallel Basic—Long	0.96	0.93	0.94	356
Parallel Basic—Short	0.92	0.84	0.88	231
Parallel Dynamic—Long	0.84	0.94	0.89	269
Parallel Dynamic—Short	0.95	0.94	0.94	241
Accuracy			0.92	1097
Macro avg	0.92	0.91	0.91	1097
Weighted avg	0.92	0.92	0.92	1097

**Table 7:** Comparison to the SOTA and baselines. A comparison of our supervised baseline (STL), multi-task learning with shared encoder and decoder (CAE), and the proposed multi-task learning multi-channel AE (MCAE) with the state of the art based on F1 score.

	Model	UCI-HAR	MHealth	PAMAP2	USC-HAD	Alpine Skiing
SOTA HAR Models	Zhang et al. [53]	98.42	-	-	-	-
	Abedin et al. [47]	-	-	90.08	-	-
	Li et al. [49]	-	-	97.35	-	-
	Zhang et al. [54]			99.00	86.00	-
	Sena et al. [48]	-	93.49	75.82	80.65	-
	Auh et al. [43]	-	96.37	85.85	-	-
	Abbaspour et al. [44]	-	-	95.12	-	-
	Tong et al. [50]	95.45	-	-	-	-
	Ek et al. [51]	97.67	-	-	-	-
Single Task Learning	Supervised Baseline (STL)	97.53	99.23	92.66	76.17	87.64
Multi-Task Learning	Classical AE (CAE)	94.61	97.33	91.00	75.69	62.08
	**Multi-Channel AE (MCAE)**	98.55	99.58	94.88	83.90	91.24

**Table 8:** A comparison of F1 score, multi-task learning with our proposed multi-channel AE (MCAE) trained by Selu and Relu, and multi-task learning with shared encoder and decoder (CAE).

Model	UCI-HAR	MHealth	PAMAP2	USC-HAD	Alpine Skiing
Ours with Selu	98.55	99.58	94.88	83.90	91.24
Ours with Relu	95.48	99.93	93.95	71.64	86.88
CAE	94.61	97.33	91.00	75.69	62.08

**Table 9:** A comparison in terms of the number of parameters and F1 score on the UCI-HAR and PAMAP2 datasets. Reported values by [46,49] are weighted F1 and accuracy, respectively. M: Million, K: Thousand.

Model	UCI-HAR		PAMAP2	
	Number of Parameters	F1 Score	Number of Parameters	F1 Score
Ours	90 K	98.55	258 K	94.88
CAE	9.7 K	94.61	29 K	91.00
Tong et al. [50]	1.1 M	95.45	-	-
Ek et al. [51]	1.27 M	97.67	-	-
Li et al. [49]	-	-	185 K	97.35
Gao et al. [46]	-	-	3.51 M	93.16

## Data Availability

Data are contained within the article.
